# Incidence and predictors of recurrent stroke in patients with cryptogenic stroke later diagnosed with atrial fibrillation: a 5-year cohort study from a tertiary care hospital

**DOI:** 10.1097/MS9.0000000000004631

**Published:** 2026-01-21

**Authors:** Saad Ali, Ikram Ullah, Sadiq Ali Shah, Sher Wali Khan, Shakir Ullah, Samiullah Khan, Ziaillah Qazi, Ayesha Fayyaz, Zubair Ahmad, Naqeeb Ullah, Sardar Noman Qayyum, Rafiullah Hotak, Ramin Khan, Muhammad Rehan, Irfan Ullah

**Affiliations:** a Department of Neurtology, MTI Lady Reading Hospital, Peshawar, Pakistan; b Department of Cardiology, Institute of Basic Medical Sciences Peshawar, Pakistan; c DHQ hospital Timergira, Pakistan; d Department of Medicine, SUNY Downstate Medical Center, State University of New York Downstate Health Sciences Center, Brooklyn New York, US; e Resident internal Medicine, Hayatabad Medical Complex, Peshawar, Pakistan; f Resident internal medicine, Lady Reading Hospital, Peshawar, Pakistan; g Bacha Khan Medical College, Mardan, Pakistan; h Al-Nafees Medical College, Islamabad, Pakistan

**Keywords:** anticoagulation, atrial fibrillation, cardiac monitoring, cryptogenic stroke, recurrent stroke, South Asia

## Abstract

**Background:**

Cryptogenic stroke (CS) accounts for a substantial proportion of ischemic strokes, with atrial fibrillation (AF) often identified as the underlying cause. In countries with low-resource settings like Pakistan, limited access to extended cardiac monitoring delays AF detection, increasing the risk of recurrent stroke.

**Objective:**

To determine the incidence and predictors of recurrent ischemic stroke in patients initially diagnosed with CS and later diagnosed with AF at a tertiary care hospital in Pakistan.

**Methods:**

A retrospective cohort study was conducted at tertiary care Hospital, from January 2018 to December 2023. Adult patients with CS and a subsequent diagnosis of AF within 12 months were included. Data on demographics, diagnostic modality, echocardiography, anticoagulation adherence, and outcomes were collected. Cox regression was used to identify independent predictors of recurrent stroke.

**Results:**

Out of 406 patients with CS, 109 (26.8%) were later diagnosed with AF. Recurrent ischemic stroke occurred in 32 patients (29.4%) over a median follow-up of 13.8 months. Only 11 of the recurrent cases (34.4%) were on anticoagulation therapy at the time of recurrence. Multivariate analysis identified age >65 years (HR: 2.21; 95% CI: 1.18–4.14), delayed AF detection beyond 6 months (HR: 2.58; 95% CI: 1.36–4.91), non-adherence to anticoagulation (HR: 3.44; 95% CI: 1.88–6.27), and left atrial enlargement (HR: 2.03; 95% CI: 1.15–3.57) as independent predictors of recurrence. Mortality (18.8%) and functional disability (mRS >3 in 59.3%) were significantly higher in the recurrence group.

**Conclusion:**

Delayed AF detection and poor anticoagulation adherence significantly increase the risk of recurrent stroke in CS-AF patients. Prolonged cardiac monitoring and improved access to anticoagulants, particularly NOACs, are essential to reduce recurrent stroke burden in low-resource settings.

## Introduction

Stroke remains a global public health priority ranking as the second leading cause of death, and a major cause of disability worldwide^[^1^]^. In 2021, an estimated 11.9 million people suffered a new stroke (index stroke event). Low- and middle-income countries have a disproportionate share of this burden, accounting for about 87% of stroke deaths^[^1^]^. South Asia in particular faces a growing stroke incidence and mortality, with patients often affected at a younger age compared to Western population^[^2^]^. Pakistan is no exception with stroke being the leading cause of adult disability, yet the epidemiological data from this region are limited. Two small community-based studies in Karachi reported stroke prevalence rates of 4.8 and 19.1%^[^2^]^.

HIGHLIGHTS

- Atrial fibrillation (AF) detected in 26.8% of cryptogenic stroke patients within 12 months.
- Recurrent ischemic stroke occurred in 29.4% of CS-AF patients.
- Non-adherence to anticoagulation tripled recurrence risk
- Older age and left atrial enlargement predicted recurrence
- Early AF detection reduced recurrent stroke burden

About one in four ischemic strokes is classified as cryptogenic, which is defined as stroke with no known etiology despite a thorough diagnostic workup^[^3^]^. Cryptogenic Stroke (CS) is a brain infarction not attributable to any established etiologies, including large artery atherosclerosis, small vessel occlusion, or a cardioembolic source. It represents a diagnostic challenge. In many cases, an occult embolic mechanism is suspected, leading to the concept of embolic stroke of undetermined source (ESUS) as a subset of cryptogenic stroke^[^4^]^. Potential hidden causes of CS include subclinical atrial fibrillation (AF), patent foramen ovale (PFO), aortic arch atherosclerosis, and other covert prothrombotic conditions. Distinguishing among these possibilities is difficult, and standard stroke evaluations often fall short of uncovering an underlying etiology.

As a result, CS patients are typically managed with antiplatelet therapy by default, even though a proportion may harbor an undetected cardioembolic source treatable with anticoagulation. The prevalence of cryptogenic stroke in most populations is substantial, 20–30% of the ischemic strokes^[^5^]^ translating to a large cohort of patients in whom optimal secondary prevention is uncertain. A leading hypothesis is that AF is an underdiagnosed cause of cryptogenic stroke. AF is a well-established risk factor for ischemic stroke, increasing stroke risk fivefold, and accounting for 20–30% of all ischemic strokes^[^3,6^]^. Importantly, strokes related to AF tend to be more severe and are associated with higher rates of long-term disability and mortality than non-AF strokes^[^6^]^.

This is attributable to AF-related strokes often causing large, thromboembolic occlusions. Early detection of AF is therefore critical, as it allows initiation of anticoagulation which dramatically reduces stroke risk. However, AF often poses a diagnostic challenge because it can be paroxysmal and asymptomatic. Up to 40% of the AF patients including those with paroxysmal episodes may never experience palpations or other symptoms^[^7^]^. These subclinical AF episodes can cause stroke recurrence in the stroke patients. In fact, cryptogenic stroke may be the first manifestation of previously unrecognized AF in many patients. It is also suspected that an episode of paroxysmal AF that terminates spontaneously can lead to cardiac thrombus formation and embolic stroke, then remain undetected during routine evaluations^[^8^]^.

Standard cardiac monitoring performed during acute stroke hospitalization has well-known limitations for detecting paroxysmal AF. In many stroke units, patients receive only a 24–48-hour period of ECG or Holter monitoring. Unfortunately, this duration is often insufficient to capture an intermittent arrhythmia. Studies have shown that a single 24–48-hour Holter detects paroxysmal AF in only about 3–5% of cryptogenic stroke patients. Multiple strategies for prolonged rhythm surveillance have been evaluated in cryptogenic stroke. These include repeated Holter monitoring, long-term ambulatory ECG devices, and implantable loop recorders (ILRs)^[^9^]^.

The early detection of AF after a cryptogenic stroke carries profound therapeutic implications for secondary prevention. Once AF is reported, the patient’s stroke is reclassified as a cardioembolic stroke and oral anticoagulation (OAC) becomes the indicated preventive strategy. Anticoagulation with warfarin or direct oral anticoagulants can reduce the risk of recurrent stroke by approximately two thirds in patients with AF, compared to anti-platelet therapy or no therapy. In practical terms, timely identification of AF allows physicians to initiate highly effective stroke prevention with OAC, which has been shown to dramatically lower the risk of future ischemic events in AF patients. Conversely, if AF is not detected, a cryptogenic stroke patient will typically remain on antiplatelet monotherapy, which may be insufficient if the true stroke mechanism was cardioembolic. Recent prospective studies suggest that cryptogenic stroke patients whose AF is detected and treated do *not* have a higher recurrence rate than other stroke patients, presumably because anticoagulation mitigates their risk^[^10^]^.

Despite the global advancements in the understandings of CS and AF, there is evidence of a gap in South Asia especially from Pakistan. Hospital-based studies can bridge that gap in the absence of nationwide stroke registries. Therefore, in this retrospective study, we aimed to provide one of the first detailed analyses of CS patients with AF from this region. We used the STROCCS 2025 guidelines to report the study findings^[^11^]^.

## Methods

### Study design and setting

A retrospective cohort study was conducted at the Neurology and Cardiology Departments of a tertiary care hospital. The study period spanned over 5 years, from January 2018 to December 2023. Ethical approval was obtained from the institutional review board prior to data collection

### Patient selection

Patients ≥ 18 years who were admitted with a diagnosis of cryptogenic ischemic stroke (CS) were identified through the hospital stroke registry. CS was defined as an acute ischemic stroke of undetermined etiology despite standard diagnostic evaluation, including neuroimaging, vascular imaging, echocardiography, and routine laboratory workup.

### Diagnosis of cryptogenic stroke

Cryptogenic stroke is diagnosed after excluding all identifiable causes through a stepwise workup that typically includes CT followed by MRI with DWI to define infarct pattern, CTA/MRA or carotid Doppler to rule out significant stenosis or dissection, and cardiac evaluation with ECG, TTE, and where available TEE to detect structural or embolic sources. When vascular imaging, cardiac studies, rhythm monitoring for 24–72 hours, and targeted labs reveal no clear etiology, the stroke is classified as cryptogenic.

### Inclusion and exclusion criteria

The study’s inclusion criteria included the patients with an initial diagnosis of CS and subsequent diagnosis of AF within 12 months of the index stroke, confirmed via 12-lead electrocardiogram, 24-hour Holter monitoring, or implantable loop recorder (ILR). Whereas patients with known history of AF or atrial flutter prior to the index stroke, and patients with structural heart diseases, valvular abnormalities, or congenital cardiac anomalies were excluded. Patients with incomplete clinical or follow-up data were also excluded.

### Data collection

Data were collected from the medical records and stroke registry. Variables collected included: Demographic characteristics including age, and gender, vascular risk factors such as hypertension, diabetes mellitus, smoking status, and prior transient ischemic attack (TIA), stroke severity on admission, diagnostic modality used for AF detection (ECG, Holter, and ILR), and time to diagnosis, Echocardiographic data, including left atrial measurements, treatment data including anticoagulation initiation and adherence. The clinical outcomes such as recurrent ischemic stroke, time to recurrence, mortality and disability (as assessed by modified Rankin Scale) were also captured. Anticoagulation adherence was assessed via documented follow-up visits, and left atrial enlargement was defined based on echocardiographic reference standards.

### Outcomes

The primary outcome was the occurrence of a recurrent ischemic stroke during the follow-up period. Secondary outcomes included: time from index stroke to AF diagnosis, time from AF diagnosis to recurrent stroke, all causes of mortality during follow-up, and functional disability at last follow-up.

### Statistical analysis

Data were analyzed using SPSS version 27. The categorical variables were summarized by frequencies and percentages, and compared using Chi-square, while continuous variables were presented as mean ± standard deviation. Cox proportional hazard regression modeling was performed to identify independent predictors of recurrent stroke. Hazard ratios (HR) and 95% confidence intervals (CIs) were reported. A *P*-value of < 0.05 was considered as statistically significant.

## Results

### Patient demographics

A total of 406 patients presented with cryptogenic ischemic stroke (CS) between January 2018 and January 2023. Among them 109 patients (26.8%) were subsequently diagnosed with AF within 12 months of their index stroke and met the inclusion criteria. The mean age of this cohort was 63.4 ± 11.2 years, and 66 patients (60.5%) were male. The median time to AF diagnosis was 5.5 months [Interquartile range (IQR): 3.2 to 8.9 months]. AF was detected using 24-hour Holter monitoring in 52 patients (48%), 12-lead electrocardiogram (ECG) in 43 patients (39%), and implantable loop recorders (ILRs) in 14 patients (13%). Table 1 summarizes the baseline characteristics of the patients.

**Table 1:** Demographic characteristics of the patients

Variable	*N* (%)
Total CS patients	406
Patients with AF diagnosis	109 (26.8%)
Mean age	63.4 ± 11.2
Male	66 (60.5%)
Median time to AF detection	5.5 months
Detection method – Holter	52 (48%)
Detection method – ECG	43 (39%)
Detection method – ILR	14 (13%)

Demographic and baseline characteristics of cryptogenic stroke (CS) patients diagnosed with atrial fibrillation (AF). Data are presented as mean ± standard deviation (SD) or number (percentage).

### Recurrent stroke and clinical outcomes

Among the 109 patients diagnosed with AF, 32 (29.4%) experienced a recurrent ischemic stroke during the follow-up period. The median time to recurrent stroke was 13.8 months (IQR: 8.6 to 21.4 months) as shown in Table 2. Notably, only 11 of these 32 patients (34.4%) were on anticoagulation therapy at the time of recurrence. Patients who experienced recurrent stroke had significantly worse clinical outcomes compared to those who remained recurrence-free during the follow-up period. The mortality rates were 18.8 and 6.0% in the recurrence and non-recurrence group respectively (*P* = 0.041), and functional disability (modified Rankin Scale [mRS] score > 3 at final follow-up) was reported in 59.3% of the patients in the recurrence group compared to 28.9% in the non-recurrence group (*P* = 0.008) as shown in Table 3.

**Table 2:** Recurrent stroke outcomes

Outcome	Value
Recurrent stroke	32 (29.4%)
Median time to recurrence	13.8 months
Deaths in recurrence group	6 (18.8%)
mRS >3 at final follow-up	19 (59.3%)
Anticoagulated at recurrence	11 (34.4%)

Clinical outcomes of cryptogenic stroke (CS) patients diagnosed with atrial fibrillation (AF) who experienced recurrent ischemic stroke. Data are presented as number (percentage) or median (months).

**Table 3:** Clinical outcomes comparison

Outcome	Recurrent group	Non-recurrent group	*P*-Value
Mortality	6 (18.8%)	5 (6.0%)	0.041
mRS >3 at final follow-up	19 (59.3%)	22 (28.9%)	0.008

Comparison of clinical outcomes between cryptogenic stroke (CS) patients with and without recurrent ischemic stroke. Outcomes include mortality and functional disability [modified Rankin Scale (mRS) > 3].

### Predictors of recurrent stroke

Multivariate Cox proportional hazards analysis identified the following as independent predictors of recurrent stroke: age > 65 years, AF diagnosis post 6 months of the index stroke, no-adherence to anticoagulation therapy and left atrial enlargement were assessed as independent predictors of recurrent stroke. Non-adherence to anticoagulation emerged as the strongest predictor of recurrence with threefold increased risk. Table 4 summarizes the predictors of recurrent stroke.

**Table 4:** Predictors of recurrent stroke

Predictor	HR	95% CI	*P*-Value
Age > 65 years	2.21	1.18–4.14	0.011
AF diagnosis >6 months	2.58	1.36–4.91	0.003
Non-adherence to anticoagulation	3.44	1.88–6.27	<0.001
Left atrial enlargement	2.03	1.15–3.57	0.016

Predictors of recurrent ischemic stroke among cryptogenic stroke (CS) patients diagnosed with atrial fibrillation (AF). Hazard ratios (HRs) with 95% confidence intervals (CIs) are presented.

## Discussion

The findings of this cohort study add to the growing body of evidence on stroke and AF. We found that a substantial proportion of cryptogenic stroke patients (26.8%) had AF, consistent with a Japanese multicenter study which found that 32.7% of CS patients had been diagnosed with AF over 24 months using ICMs^[^12^]^. A meta-analysis of 59 studies also reported an average post-stroke AF detection rate of 20.8%^[^13^]^. Many single center cohorts in the West report even higher yields with prolonged monitoring; an Italian real-world study demonstrated AF in 41% of CS patients followed for 30 months with ICMs^[^3^]^. For instance, an Indian study has found lower (only 11%) AF incidence at 1 year follow-up^[^14^]^. These differences likely reflect patient age, duration, and technique of monitoring employed.

Our 5-year retrospective cohort from a tertiary care facility reported a 29.4% incidence of recurrent ischemic stroke in patients initially diagnosed with CS with subsequent diagnosis of AF. This recurrence rate aligns with long-term data from other populations, for example, a community-based cohort in the United States observed approximately one-third cryptogenic stroke patients experiencing recurrence over 5-year^[^15^]^. Studies in Asia reported annual recurrence risks around 4–10% at 1-year follow-up^[^16^]^. Notably recurrence rates appear to be dependent on secondary prevention strategies. In cohorts, where AF is promptly diagnosed and treated, the risk of recurrent stroke can be significantly lowered^[^12^]^. For instance, a recent Japanese multicenter study of CS patients monitored with insertable cardiac monitors (ICMs) found only a 5.8% stroke recurrence rate over a 1.5-years in those with AF detected and anticoagulation therapy started, comparable to the 4% in patients without AF^[^12^]^. This similarity suggests that when AF is identified and managed, cryptogenic stroke patients do not suffer disproportionately higher recurrences than other stroke subtypes. By contrast, the high 29.4% recurrence in our cohort likely reflects cases where AF went untreated or underdiagnosed for significant periods, indicating the consequences of missed or delayed diagnosis and suboptimal anticoagulation in a real-world South Asian setting.

The median time to AF detection in our cohort was 5.5 months, indicating that most of the times AF was discovered within the first half-year after the index score. International stroke guidelines recommend at least 72 hours of rhythm monitoring after CS, as longer and earlier cardiac monitoring substantially increases AF detection yield^[^15^]^. Resource-intensive methods like ILRs offer the highest yield: pooled data show that roughly 25–30% of CS patients have AF detected within 3 years of ILR monitoring (median time to detection 7.9 months)^[^17^]^. For instance, a Pakistani study using a 48-hour Holter monitoring in young stroke patients detected paroxysmal AF in 16.7% of the cases^[^18^]^ yet another reported an initial AF detection of 25% with a single 48-hour Holter. These findings reinforce that prolonged cardiac monitoring is critical to uncover occult AF in CS patients^[^19^]^. Early detection of AF (within the first 3 months) appeared beneficial as it allowed timely initiation of therapy and was associated with a significantly prolonged time to any stroke recurrence in one study^[^20^]^. Conversely, if AF detection is delayed or missed, patients remain essentially in an untreated, high-risk state, which can precipitate earlier recurrence^[^20^]^. Therefore, South Asian practices should incorporate extended monitoring (72-hour Holder or beyond) and prioritize high-risk patients for ILRs, given the prevalence of CS in the relatively young stroke population in the region.

We found that only 34.4% of the patients in the recurrence group were on therapeutic anticoagulation at the time of their repeat stroke, implying that nearly two-thirds were either not on the anti-coagulation therapy or not adherent. Non-adherence to anti-coagulation also emerged as the strongest independent predictor of recurrent stroke in our analysis (HR 3.44). This aligns with the broader evidence that appropriate anticoagulation is the single most effective intervention to prevent stroke recurrence in AF patients, reducing recurrence risk by roughly 60%^[^15^]^. AF related strokes tend to be more severe and debilitating than other ischemic stroke subtypes. Prior studies have found that strokes in patients with AF have nearly double the early mortality odds compared to non-AF strokes^[^15^]^. Effective reperfusion therapies also tend to have reduced success in AF patients, partly due to older age, and comorbidities clustering with AF. Recurrent strokes also compound the risk, a second stroke often adds to disability and can be fatal, especially if the patient was already neurologically or medically affected from the first event. A recent Indian ESUS study reported over 70% of the patients had a modified Rankin Scale (mRS) score ≤ 2 at 12 months^[^16^]^. However, recurrent stroke invariably affects this recovery. We found that patients with recurrent strokes would have a higher degree of disability and mortality compared to those without recurrence. AF detection and anticoagulation not only prevents stroke recurrence but also spares the patient from potential worsening of mRS and reduces the substantial risk of stroke-related death.

We found several predictors of recurrent stroke in the CS patients diagnosed with AF. Older patients not only are more likely to have occult AF detected after a CS, but also tend to have more aggressive strokes^[^20^]^. Time of AF detection is another critical factor. Early AF detection appears to delay or prevent stroke recurrence, whereas undetected AF leaves a patient exposed. Xu *et al*^[^20^]^ observed that among CS patients who suffered a recurrence, those whose AF was discovered early had their recurrent strokes much later compared to those whose AF was found after a recurrence. This temporal association implies that prompt identification of AF can prolong the recurrence-free interval, likely by enabling timely anticoagulation. We also identified left-atrial enlargement as a significant predictor of recurrence. It was also identified as an independent predictor of AF in CS patients earlier in a study^[^20^]^. LA enlargement reflects an underlying atrial myopathy that predisposes the patient to fibrillation as it may also indicate a greater propensity for embolus formation in the left atrium.

### Implications and limitations

This hospital-based retrospective study highlights a significant recurrence risk (29.4%) in CS patients later diagnosed with AF. The study findings reinforce the importance of early detection and adherence to anticoagulation, especially in resource-limited South Asian settings. The study also identified predictors such as age > 65 years, delayed AF detection, left atrial enlargement, and non-adherence can guide risk stratification and monitoring strategies. However, being a single-center retrospective study, results may not be generalizable. Variable AF monitoring methods, and incomplete follow-up could have led to underdiagnosis. Data on anticoagulation type, INR control and reasons for non-adherence were limited. Functional outcomes were based solely on mRS.

## Conclusion

This retrospective hospital-based study from a tertiary care hospital highlights the significant risk of recurrent ischemic stroke among patients initially diagnosed with CS with subsequent diagnosis with AF. Nearly one-third experienced recurrence and anticoagulation nonadherence emerged as the strongest predictor. Age > 65 years, delayed AF diagnosis and left atrial enlargement further stratify recurrence risk. These results call for implementation of guideline-based cardiac monitoring, improved access to anticoagulation therapy, and robust patient follow-up.

## Data Availability

The dataset used and analyzed during the current study are available from the corresponding author, Ikram Ullah, upon reasonable request.

## Research registration unique identification number (UIN)

This study is registered on ClinicalTrials.gov under the identifier NCT07126184. The full trial details are publicly accessible at: https://clinicaltrials.gov/study/NCT07126184.
